# Monitoring Regulatory Immune Responses in Tumor Immunotherapy Clinical Trials

**DOI:** 10.3389/fonc.2013.00109

**Published:** 2013-05-06

**Authors:** Brian M. Olson, Douglas G. McNeel

**Affiliations:** ^1^Department of Medicine, University of Wisconsin Carbone Cancer CenterMadison, WI, USA

**Keywords:** regulatory T cells, myeloid-derived suppressor cells, immune monitoring, tolerance, suppression, predictors of response

## Abstract

While immune monitoring of tumor immunotherapy often focuses on the generation of productive Th1-type inflammatory immune responses, the importance of regulatory immune responses is often overlooked, despite the well-documented effects of regulatory immune responses in suppressing anti-tumor immunity. In a variety of malignancies, the frequency of regulatory cell populations has been shown to correlate with disease progression and a poor prognosis, further emphasizing the importance of characterizing the effects of immunotherapy on these populations. This review focuses on the role of suppressive immune populations (regulatory T cells, myeloid-derived suppressor cells, and tumor-associated macrophages) in inhibiting anti-tumor immunity, how these populations have been used in the immune monitoring of clinical trials, the prognostic value of these responses, and how the monitoring of these regulatory responses can be improved in the future.

## Introduction

Recent years have seen several exciting advancements in the development of active immunotherapy for the treatment of cancer. Sipuleucel-T, an active immunotherapy comprised of autologous dendritic cells (DC) pulsed with a fusion protein composed of granulocyte macrophage colony-stimulating factor (GM-CSF) and prostatic acid phosphatase (PAP), was shown to provide a significant increase in overall survival in patients with metastatic prostate cancer (Kantoff et al., 2010). Additionally, ipilimumab, an antibody blocking cytotoxic T lymphocyte-associated antigen 4 (CTLA-4) that facilitates T cell activation, was found to provide a benefit in overall survival in individuals with metastatic melanoma (Hodi et al., 2010). While the success these agents had in Phase III clinical trials represented a ground shift in our understanding of the potential of anti-tumor immunity, the results from these trials also illuminated challenges with the clinical evaluation of immunotherapies. As opposed to therapies with direct cytotoxic effects, like chemotherapy or radiation therapy, immune-modulating therapies require time to activate the immune system and induce T-cell proliferation to sufficient levels where it can achieve clinical benefit, a process which may take place over weeks to months. As such, while randomized trials evaluating sipuleucel-T and ipilimumab both achieved the clinical endpoint of increased overall survival, they were not able to meet interim markers of efficacy such as increased time to disease progression. This emphasizes the importance of identifying short-term markers of efficacy that can be used to identify individuals who are responding to therapy, or those who would benefit from moving on to alternative treatments.

As one of the central goals of tumor immunotherapy is to elicit and/or augment cytotoxic T-cell responses that can recognize and lyse tumor cells, the development of interim biomarkers of immunotherapeutic efficacy have largely focused on assays that measure these inflammatory, Th1-type anti-tumor responses. This has led to the near universal use of assays such as enzyme-linked immunosorbent spot (ELISPOT) assays, intracellular cytokine staining (ICCS), and HLA-peptide multimer analysis. However, as our understanding of the nature of the relationship between the tumor and immune response has matured, tumor immunologists have come to appreciate that these effector responses are only one aspect of the immune system that can impact anti-tumor immunity. The immune system (and the tumor itself) is also able to mount suppressive immune responses that target effector responses and can lead to the amelioration of anti-tumor responses. These suppressive immune responses are predominantly composed of regulatory T cells, myeloid-derived suppressor cells (MDSCs), and tumor-associated macrophages (TAM), which are able to survey the tumor microenvironment for effector immune responses to inhibit, which leads to the avoidance of anti-tumor immunity and further tumor growth. The monitoring of changes in regulatory immune responses, consequently, could theoretically serve as an additional biomarker of response to immune therapies, particular in the case of immune-modulating therapies or whole tumor vaccines where a specific antigenic target is unknown.

While there has been interest in monitoring suppressive immune responses following immunotherapeutic intervention, this has been a challenge given the difficulty in defining a set of cellular surface markers that can be used to easily identify and quantify regulatory immune responses. Furthermore, when evaluating antigen-specific vaccine approaches, there has been a noticeable paucity in the evaluation of antigen-specific regulatory responses following immunization. In addition, the intrinsic plasticity in regulatory immune function further complicates this analysis, as data in preclinical models indicates that immune responses can gain and lose suppressive activity depending on the microenvironment, which is particular important in the case of lymphocytes that infiltrate the suppressive tumor microenvironment. In this review, we will describe these regulatory cell populations, how they can suppress anti-tumor immune responses, how they have been used in the immune monitoring of clinical trials, and challenges associated with the implementation of regulatory cell detection into clinical trial immune monitoring.

## Immune Regulatory Populations and Their Effects on Cancer

### Regulatory T cells

Regulatory T cells (Tregs) are a subset of T lymphocytes identified in the early 1970s (Gershon and Kondo, 1970) that have the ability to suppress the activity of effector T cells. In healthy individuals, Tregs play a crucial role in maintaining peripheral immune tolerance, preventing the generation of autoimmunity by limiting T-cell activity. However, in the case of malignant disease, these cells can act to limit anti-tumor immune responses and confound the efforts of immunotherapeutic approaches. A variety of malignancies have been shown to have increased frequencies of both peripheral and tumor-infiltrating Tregs, including patients with lung, pancreas, ovarian, breast, and prostate cancer (Woo et al., 2001, 2002; Liyanage et al., 2002; Miller et al., 2006; Kiniwa et al., 2007; Pages et al., 2010). Additionally, the detrimental impact Tregs can have on anti-tumor responses is further suggested by their prognostic value, as higher frequencies of Tregs correlate with disease stage and poor prognoses in a variety of malignancies (Curiel et al., 2004; Beyer et al., 2005; Wolf et al., 2005; Betts et al., 2006; Hiraoka et al., 2006; Kono et al., 2006; Kobayashi et al., 2007; Akin et al., 2011; Katz et al., 2013).

These regulatory responses are broadly broken up into either “natural” or “adaptive/induced” Tregs. Natural Tregs (nTregs) are produced by the thymus and constitutively express CD25, CTLA-4, and Foxp3 (a transcription factor that helps mediate the suppressive activity of this regulatory population), and are able to suppress both adaptive and innate immune responses (Read et al., 2000; Takahashi et al., 2000; Mougiakakos et al., 2010). These cells are generated in the thymus by the selection of thymocytes that have T-cell receptors with high avidity for self-antigens – thus, nTregs are responsible primarily for maintaining self-tolerance (Jordan et al., 2001). They are able to maintain this self-tolerance through a variety of mechanisms including the secretion of inhibitory cytokines (such as IL-10, TGF-β, and IL-35), direct cytotoxicity, disruption of T-cell metabolism, or targeting the activity of DC through inhibitory surface molecules such as CTLA-4 or LAG-3 (Vignali et al., 2008).

As opposed to nTregs, induced Tregs (iTregs) enter the periphery as naïve T cells. However, rather than gain an effector phenotype, these iTregs encounter their specific MHC-peptide complex under conditions that promote the development of a regulatory phenotype, such as high levels of suppressive cytokines (Lohr et al., 2006). This is particularly relevant to tumor immunology, as the tumor microenvironment is saturated with factors that can promote the generation of iTregs, including factors that are directly produced by tumor cells such as TGF-β, IL-10, IL-35, and indoleamine 2,3-dioxygenase (IDO; Liu et al., 2007; Collison et al., 2010; Heckel et al., 2011; Wang et al., 2013). This has led to numerous reports of lymphocytes which infiltrate tumors as effector cells, but are then converted to have a regulatory phenotype, further confounding the efforts of tumor immunologists to generate productive anti-tumor immunity (Valzasina et al., 2006; Liu et al., 2007; Shafer-Weaver et al., 2009; Collison et al., 2010).

Inducible Tregs are further subdivided into Tr1, Th3, and Tr35 cells, which are loosely divided based on their mechanisms of suppression. Tr1 cells rely largely on IL-10 secretion to mediate suppression, are developed in the presence of high doses of IL-10, and express very low or no Foxp3 and CD25 (Groux et al., 1997; Roncarolo et al., 2001; Levings et al., 2005). Th3 cells produce high levels of TGF-β to mediate suppression, and contrary to Tr1 cells, also express CD25 and Foxp3 (Chen et al., 1994; Weiner, 2001). The final population, iTr35 cells, is a population of induced regulatory T cells that rely on IL-35, a suppressive cytokine that was identified as having the ability to potently suppress T-cell proliferation and T-cell induced autoimmunity and anti-tumor responses (Collison et al., 2007, 2010). One of the hallmarks of iTr35 cells is that while they do not express Foxp3, their production of IL-35 has the ability to convert conventional T cells, nTreg, and other iTreg populations into iTr35 cells, resulting in mixed expression of Foxp3 and CD25 (Collison et al., 2010). As a result, while it can be difficult to identify iTr35 cells based on surface molecule expression, these iTr35 cells have a significant ability to propagate infectious tolerance [the transfer of suppressive function from one cell to another (Gershon and Kondo, 1971; Qin et al., 1993)].

While these natural and induced regulatory T cells are conventionally viewed as CD4+ T cells, some of the earliest work into suppressive T cells identified that CD8+ T cells could also mediate the suppression of immunity *in vivo* (Gershon and Kondo, 1970). This has been reinforced by research in the last decade, with the identification of CD8+ T cells with potent suppressive activity (Cortesini et al., 2001; Sarantopoulos et al., 2004; Wei et al., 2005; Chaput et al., 2009; Olson et al., 2012). These CD8+ regulatory T cells can also be divided into natural and induced regulatory T cells, which can also mediate suppression by contact-dependent and -independent mechanisms. However, our understanding of the nature of these CD8+ regulatory T cells remains mercurial and a topic of continued investigation.

While research characterizing regulatory cells has made significant progress, one of the challenges that have come to light from these studies is the difficulty in defining a phenotype that can be used to reliably identify a regulatory T cell. Common markers used to identify Tregs are CD25, Foxp3, CD39, CD122, CD127, CTLA-4, LAG-3, and GITR (Mougiakakos et al., 2010). The expression of CTLA-4 is especially important, as therapies designed at blocking the activity of Tregs have been developed that specifically target this molecule. However, each of these markers can be expressed by other T cell subtypes, including activated effector T cells, preventing them from being used as exclusive markers associated with Tregs. Furthermore, CD4+ Tr1 cells have negligible levels of CD25 and Foxp3, further complicating a complete analysis of Tregs.

While research studying regulatory T cells has focused on antigen non-specific populations, emerging evidence has also shown a role for antigen-specific regulation in cancer. These antigen-specific Tregs require their cognate antigen to activate their suppressive activity; however, once active, these cells can suppress in an antigen non-specific fashion, so-called “bystander suppression” (von Herrath and Harrison, 2003). CD4+ regulatory T cells have been identified that are specific for a variety of tumor antigens, including antigens commonly targeted by vaccination such as gp100, NY-ESO-1, HER2/neu, and CEA (Wang et al., 2004, 2005; van der Burg et al., 2007; Vence et al., 2007; Lehe et al., 2008; Welters et al., 2008; Bonertz et al., 2009; Mougiakakos et al., 2010). Additionally, antigen-specific CD8+ T cells with suppressive function have also been identified (Andersen et al., 2009; Olson et al., 2012). We recently identified CD8+ suppressor T cells that were present in peripheral blood samples from patients with prostate cancer that were specific for PAP (the antigen targeted by sipuleucel-T), and prevented the detection of effector responses following vaccination with a DNA vaccine targeting PAP (Olson et al., 2012).

### Myeloid-derived suppressor cells

Myeloid-derived suppressor cells are a diverse population of myeloid cells which have been shown to have the ability to suppress the proliferation and effector function of T cells. MDSCs consist primarily of immature myeloid cells and myeloid progenitor cells, cells which have not finished their differentiation into DCs, macrophages, or granulocytes. In healthy individuals, MDSCs represent a very small fraction of total peripheral blood mononuclear cells (PBMC), as these immature cells rapidly differentiate into mature cells. In a variety of malignancies, however, this differentiation process is blocked, leading to the generation of a sizable fraction of MDSCs. This is true in patients with many types of cancer, including lung, breast, colon, and melanoma, where patients have an increased frequency of peripheral and tumor-infiltrating MDSCs, and in some cases these frequencies correlate with disease grade (reviewed in Montero et al., 2012).

Studies in mouse models have identified two categories of CD11b+ MDSCs based on their expression of the myeloid differentiation antigen Gr1 (which recognizes the Ly6G and Ly6C epitopes) – granulocytic MDSCs (CD11b+Ly6G+Ly6C^low^) and monocytic MDSCs (CD11b+Ly6G−Ly6C^hi^). However, as humans lack a Gr1 homolog, the phenotypic characterization of human MDSCs has proved more complicated. While several surface molecules have been used to delineate MDSC subpopulations, common markers used to identify these subtypes include CD14 and CD15, with human granulocytic MDSCs being CD11b+CD33+CD14−CD15+ and monocytic MDSCs being CD11b+CD33+CD14+CD15−. Other markers that can be used in combination to identify MDSCs include CD13+, CD34+, IL-4Rα+, and HLA-DR− (Peranzoni et al., 2010).

Myeloid-derived suppressor cells can mediate the suppression of effector immune responses using a variety of mechanisms (Gabrilovich and Nagaraj, 2009). One of the most common mechanisms of suppression is focused on disrupting T-cell metabolism. This includes the production of arginase and IDO (which can deplete arginine and tryptophan, each of which are required for T-cell activity) (Mellor and Munn, 2004; Bronte and Zanovello, 2005). Additionally, MDSCs have been shown to express inducible nitric oxide synthase (iNOS), which leads to the generation of nitric oxide (NO), as well as producing reactive oxygen species (ROS), both of which can target T-cell function (Mazzoni et al., 2002; Kusmartsev et al., 2004). Research has suggested that the production of these two molecules may demarcate subtypes of MDSCs, with monocytic MDSC producing NO and granulocytic MDSC producing ROS (Movahedi et al., 2008). MDSC can also produce peroxynitrite, which can result in T-cell receptor nitration and a decrease in T-cell activity (Nagaraj et al., 2007). In addition to these mechanisms that directly target the activity of effector T cells, MDSCs have also been shown to induce the expansion of regulatory T cells, which may be due in part to their expression of regulatory cytokines like IL-10 or TGF-β, or the inhibitory receptor CTLA-4 (Huang et al., 2006; Yang et al., 2006; Serafini et al., 2008).

### Tumor-associated macrophages

Macrophages are closely linked with the development of cancer-related inflammation. In the context of cancer, these cells are divided into either type 1 or type 2 macrophages. Type 1 macrophages (M1) have the ability to present antigens and activate T-cell responses, as well as being able to directly kill tumor cells. However, in the presence of Th2-biased cytokines such as IL-10, macrophages can be diverted to gain a type 2 phenotype. These immunosuppressive type 2 macrophages (M2) are marked by the expression of CD163 (the scavenger receptor) and CD206 (the mannose receptor), as well as traditional monocyte markers such as CD14, HLA-DR, and CD11b (Mantovani et al., 2002; Biswas and Mantovani, 2010). The tumor microenvironment promotes the generation of type 2 macrophages, as tumor cells can secrete factors (such as CCL-2) that recruit macrophages to the site of the tumor, and once there the immunosuppressive tumor microenvironment is able to drive these macrophage toward a type 2 phenotype (Bottazzi et al., 1983; Heusinkveld and van der Burg, 2011). These TAM are then able to contribute to the suppressive tumor microenvironment, expressing high levels of suppressive cytokines (such as TGF-β and IL-10), promoting tumor angiogenesis, and inhibiting anti-tumor immunity (Vasiljeva et al., 2006; Coffelt et al., 2009). The detrimental impact of these type 2 macrophages is illustrated by their elevated frequency in a variety of cancers and the correlation between high frequencies and poor patient prognosis (as reviewed in Heusinkveld and van der Burg, 2011).

## Regulatory Cell Monitoring with Immunotherapy Clinical Trials

### Regulatory cell populations – biomarkers of immune and clinical response to treatment

Given the importance of regulatory immune responses in the development and progression of a variety of cancers, there has been interest in characterizing the effects of immunotherapies on the frequency of suppressive immune populations. Reports to date have predominantly focused on evaluating the effects of these therapies on regulatory T cells (particularly CD4+CD25+Foxp3+Tregs), though the effects of immunotherapies on MDSC frequency has begun to be implemented in clinical trial analyses. Most reports have focused on enumerating the frequency of Tregs following immunotherapy, with several immunotherapeutic approaches being shown to decrease the frequency of peripheral Tregs. This includes a report by Pohla et al. (2013) evaluating both Tregs and MDSCs following treatment with a allogeneic gene-modified tumor cell line in patients with metastatic renal cell carcinoma. In this study, they found that vaccination resulted in a decrease in Tregs in peripheral blood samples, which they identified using extensive phenotype analysis (CD4+CD25^hi^CD127^−/lo^Foxp3+CD39+). However, when they looked for changes in MDSCs using a variety of cell phenotypes (CD14+CD124+, CD15+CD124+, Lin-HLA-DR-CD33+SSC^hi^, SSC^imm^CD14+HLA-DR-, and CD14-CD15+CD11b+), they found that vaccination did not significantly alter any of these populations. This illustrates that while vaccination can certainly impact the frequencies of suppressive immune populations, not all populations are uniformly affected by treatment, highlighting the importance of analyzing multiple regulatory populations to get a full picture of the immune response following immunization.

While many immunotherapies have been shown to alter the frequency of regulatory cells, these changes alone do not provide information on the immune and clinical efficacy of these therapies. However, as immunotherapeutic clinical trials have begun to elicit immune and clinical efficacy, it has become possible to determine how changes in regulatory populations correlate with the efficacy of these therapies, as shown in Table 1. In nearly all these studies, it was found that a decrease in the frequency of regulatory cells (predominantly Tregs, but also a few reports evaluating MDSCs) was found to correlate with enhanced clinical benefit. For example, in two Phase II trials in patients with metastatic prostate cancer, the investigators evaluated both Treg frequency and function following treatment with a viral vaccine (Gulley et al., 2010; Vergati et al., 2011). In these studies, while there was not a significant change in the frequency of Tregs (CD4+CD25^hi^Foxp3+) following immunotherapy, they found that an increase in the ratio of effector-to-regulatory CD4+ T cells (CD4+CD25− effector T cells to CD4+CD25+CD127−Foxp3+CTLA−4+ Tregs) correlated with enhanced prognosis. Furthermore, when they isolated CD4+ Tregs and analyzed their ability to suppress autologous T cell proliferation, they found that individuals with a decrease in Treg function post-immunization had an enhanced clinical prognosis (Gulley et al., 2010; Vergati et al., 2011). This illustrates a problem common in immune monitoring, especially of regulatory immune responses – the preponderance of immune monitoring focuses on the quantity of regulatory populations rather than evaluating the quality of these suppressive populations. While the enumeration of cell populations can provide some perspective as to the efficacy of immunotherapeutic interventions, it is also possible that a smaller frequency of cells (but with more potent effector activity) can be of greater significance than a high frequency of cells with poor effector functions.

**Table 1:** **Clinical trials evaluating the effect of immunotherapy on regulatory cell frequency, and correlations with immune and clinical efficacy**.

Disease type	Immunotherapy	Cell population	Effects of immunotherapy on regulatory cells and responses	Reference
Glioblastoma	DC vaccine	Treg (CD4+CD25+CD127^lo^)	Decreased frequency of Tregs correlated with enhanced survival	Fong et al. (2012)
		CD4+CTLA-4+T cells CD8+CTLA-4+T cells	Decrease in CTLA-4 expression on CD4+ and CD8+T cells correlated with enhanced survival	
Malignant glioma	DC vaccine	Treg (CD4+CD25+CD127^lo^)	Decreases in Treg frequency correlate with increased survival	Prins et al. (2013)
B-cell chronic lymphocytic leukemia	DC vaccine	Treg (CD4+CD25+Foxp3+)	Patients with clinical responses had a significant decrease in Treg frequency	Hus et al. (2008)
Non-Hodgkin lymphoma	DC vaccine	Treg (CD4+CD25+Foxp3+)	Decrease in Treg frequency correlated with clinical responses	Di Nicola et al. (2009)
Renal	DC vaccine+therapy	Treg (CD4+CD25+Foxp3+)	Non-responding patients had significantly higher expansion of Tregs compared to responding patients.	Schwarzer et al. (2012)
Sarcoma	DC vaccine + irradiation	MDSC (CD11b+CD14− CD33+)	Higher frequencies of MDSC in non-responders	Finkelstein et al. (2012)
		Treg (CD4+CD25+Foxp3+)	No correlation between changes in Tregs and responder status	
Melanoma	Neoadjuvant ipilimumab	Treg (CD4+CD25^hi^Foxp3+)	Higher frequencies of Tregs correlated with enhanced progression-free survival	Tarhini et al. (2012)
		Monocytic MDSC (HLA-DR^lo^CD14+)	No correlation between changes in MDSC and survival	
Melanoma	DC Vaccine + IL-2	Treg (CD4+CD25^hi^)	Significant decrease in Tregs in patients with clinical responses	Bjoern et al. (2011)
		Treg (CD4+CD25^hi^Foxp3+)	No correlation between changes in Treg and clinical responses	
Melanoma	APC vaccines	Treg (D4+CD25+)	Expansion of Tregs correlated with decrease in CTL frequency	Chakraborty et al. (2004)
Prostate	Viral vaccine	Treg (CD4+CD25^hi^Foxp3+)	Decrease in Treg function post-immunization correlated with enhanced prognosis, and increased Treg function correlated with poor prognosis	Gulley et al. (2010)
Prostate	Viral Vaccine	Treg (CD4+CD25^hi^Foxp3+)	Decrease in Treg function post-immunization correlated with increased overall survival	Vergati et al. (2011)
		Effector:Treg ratio (CD4+CD25−: CD4+CD25+CD127-Foxp3+CTLA-4+)	Increased effector:Treg ratio post-immunization correlated with enhanced prognosis	
Prostate	Tumor cell vaccine + ipilimumab	Treg (CD4+CD25^hi^Foxp3+)	Increases in frequency of Tregs correlated with decreased overall survival	Santegoets et al. (2013)
		Effector:Treg ratio (CD4+CD45RO+: CD4+CD25^hi^Foxp3+)	Increases in effector: regulatory T cell ratio correlated with enhanced survival	
Lung, colorectal, gastric, breast, uterine, and renal cancer	Low-dose IL-2	Treg (CD4+CD25+)	Patients with controlled disease have a decline in number of Treg cells	Lissoni et al. (2009)
		Effector:Treg ratio (CD4+:CD4+CD25+)	Patients with controlled disease have an increase in effector:Treg ratio	
Breast	Peptide vaccine	Treg (CD4+CD25+Foxp3+)	Decrease in Tregs correlated with enhanced effector immune responses	Gates et al. (2010)

The observation that decreased regulatory cells following immunotherapy correlates with enhanced clinical responses is not wholly unexpected; as Tregs have been shown to correlate with more advanced disease and poorer prognosis in many disease types, it would be logical to conclude that a decrease in these Tregs would result in a better disease outcome following immunotherapy. However, this trend is not uniform; in a clinical trial report evaluating neoadjuvant ipilimumab in melanoma patients, the authors found that this treatment resulted in an increase in circulating Treg (both CD4+CD25^hi^Foxp3+ and CD4+CD25^hi^CD39+ T cells), and that increases in these Tregs correlated with enhanced progression-free survival (Tarhini et al., 2012). Interestingly, this group also evaluated the presence of circulating monocytic MDSCs (HLA-DR^lo^CD14+), which they found decreased following therapy. This could be a result of the treatment with ipilimumab, which targets CTLA-4 and would thus be expected to specifically target the Treg population and not MDSCs. However, it again demonstrates the importance of fully analyzing the immune response following these therapies.

Another challenge associated with regulatory cell immune monitoring is that the techniques used to identify these populations can affect results. This is exemplified in the results from three studies evaluating ipilimumab in early stage clinical trials, each of which suggested different effects of ipilimumab on Treg frequency. In a Phase I trial in prostate cancer patients, treatment with ipilimumab was found to increase Treg frequency [as measured by circulating CD4+Foxp3+ T cells (Kavanagh et al., 2008)]. In another trial evaluating ipilimumab in a variety of malignancies (colon, non-Hodgkin’s lymphoma, or prostate cancer), this treatment was found to induce a long-term decrease in CD4+ Tregs (CD4+CD25+CD62L+) (O’Mahony et al., 2007). And in yet another clinical trial in which bladder cancer patients were treated with ipilimumab, it was found that treatment-induced no consistent changes in Treg frequencies (CD4+Foxp3+, CD4+Foxp3+ICOS^hi^, or CD4+Foxp3+ICOS^lo^ T cells) (Liakou et al., 2008). The discrepancy in treatment-induced effects of Tregs based on phenotype is not only seen in patients treated with ipilimumab – in a study evaluating melanoma patients receiving a dendritic cell vaccine along with IL-2, the authors observed that a decrease in CD4+CD25^hi^ T cells correlated with a clinical response; however, they also found that there was no correlation between changes in CD4+CD25^hi^Foxp3+ T cells and disease stabilization (Berntsen et al., 2010). This highlights the importance of using well-defined and universally accepted parameters to identify regulatory populations, as well as including functional analysis of regulatory activity, to obtain the most accurate reflection of how regulatory responses are being affected by immunotherapy. This is especially important considering the plasticity of T cell function, where an effector cell can gain suppressive activity (and vice-versa) depending on the immune context (Bluestone et al., 2009; Addey et al., 2011). While immune monitoring by its nature is only a snapshot in time of this plasticity, it emphasizes that a comprehensive phenotypic and functional analysis will help provide the most accurate interpretation of that moment in time.

### Regulatory cell populations – predictive biomarkers prior to treatment

While an increase in circulating regulatory cells following immunotherapy is usually associated with a poor prognosis, and increased frequencies of regulatory cells in untreated individuals portends poor prognosis, the characterization of these populations as a prospective biomarker of immunotherapeutic efficacy remains relatively untested. Some reports have found that pre-existing Treg frequency does not correlate with vaccine efficacy one way or the other (Gulley et al., 2010; Bjoern et al., 2011). However, recent reports have found correlations between the frequency of pre-existing regulatory responses and clinical responses following immunization. In a report by Santegoets et al. (2013), evaluating combined tumor cell vaccination with ipilimumab in prostate cancer patients, the authors found that elevated frequencies of CD4+CD25^hi^Foxp3+ Tregs prior to treatment correlated with decreased overall survival following treatment. As with regulatory responses following vaccination, this is not necessarily surprising; lower frequencies of Tregs in untreated patients correlate with enhanced prognosis. However, somewhat contradictory to the other results obtained, the authors also found that increased pre-treatment frequencies of CD4+CTLA−4+ T cells correlated with enhanced survival. While CTLA-4 is expressed by activated T cells, it is also constitutively expressed by nTregs, again illustrating a potential dichotomy between results based on the phenotypic definitions of regulatory cells and necessitating additional methods of detection.

While this report found that elevated levels of CD4+CD25^hi^Foxp3+ Tregs predicted for poor prognosis, other clinical trials have found opposing results. In a report by Correale et al. (2010), the authors evaluated Treg frequencies in patients with colorectal cancer who received chemoimmunotherapy. In these individuals, the authors found that increased frequencies of Foxp3+ T cells prior to treatment correlated with enhanced overall survival and progression-free survival following treatment. However, this report differs from that of Santegoets and colleagues (and most reports monitoring the effects of immunotherapy on regulatory cell populations) in that rather than measuring circulating levels of Foxp3+ T cells, they instead measured the frequency of tumor-infiltrating cells that expressed Foxp3+ using pre-treatment biopsy samples from patients. Interestingly, another report by Hamid et al. (2011) evaluating ipilimumab in patients with melanoma also found that increased expression of Foxp3 and IDO in pre-treatment biopsy samples correlated with enhanced clinical benefit. These studies demonstrate the importance of not only evaluating the frequency of circulating regulatory cells, but also quantifying the frequency of these suppressive cells that infiltrate the tumor. As immune monitoring efforts focused on measuring peripheral effector responses have been shown to not necessarily correlate with effector responses at the site of tumor, immune monitoring efforts focused on regulatory responses should perhaps also aim to evaluate the effects of immunotherapy on tumor-infiltrating suppressive cells.

### Methods of regulatory cell detection for clinical trial analysis – beyond enumeration

While the enumeration of suppressive cell populations has been the central focus of regulatory immune monitoring, quantification alone may not be sufficient, as illustrated above. Immune monitoring efforts aimed at analyzing effector immune responses do not rely solely on quantifying the frequency of effector T cells – rather, they include functional analysis to determine the activity of these responses with respect to proliferation, cytokine expression, expression of cell surface molecules, and cytolytic activity. Similarly, immune monitoring of regulatory immunity should also include functional analysis of suppressive activity. This is particularly relevant to the analysis of regulatory immune responses, given the lack of distinct phenotypic markers that can be used to identify regulatory cells. The importance of analyzing regulatory function is clearly illustrated by the previously described reports evaluating a viral vaccine in prostate cancer patients, where immunization did not induce changes in Treg frequency that were associated with clinical responses, but decreases in Treg function were associated with an enhanced prognosis (Gulley et al., 2010; Vergati et al., 2011).

To evaluate the function of Tregs, most of the studies to date have largely focused on one of two aspects of Treg activity: suppression of T-cell proliferation and Treg expression of immunosuppressive cytokines. To measure Treg suppression, peripheral blood cells are sorted to isolate a purified Treg population (usually based on CD4+CD25+ expression), and these cells are then co-incubated with autologous CD4+CD25− conventional T cells that are non-specifically activated. T-cell proliferation can then be measured using standard techniques, including thymidine uptake, or by dilution of cell-labeling dyes such as carboxyfluorescein succinimidyl ester or PKH26, in CD4+CD25− cells. These assays can also be combined with assays measuring the effect on expression of cytokines by the effector T cells, collecting supernatants and measuring cytokine secretion by enzyme-linked immunosorbent assay (ELISA).

In addition to measuring the effect of Treg on the proliferation and cytokine secretion of effector T cells, another common assay of Treg function is measuring the cytokine expression by Tregs themselves. This has typically been performed by ELISA (where isolated Treg populations are stimulated non-specifically and cytokine release into the supernatant is measured) or ICCS. Intracellular cytokine staining has the benefit of not requiring isolation of Tregs, as whole PBMC can be isolated and stimulated, following by surface staining to identify the population of interest. Furthermore, ICCS also has the benefit of permitting concurrent staining for Foxp3, helping to identify Treg populations. While suppression assays and cytokine expression are most commonly used to evaluate Treg function, other methods employed to monitor Treg function in clinical trials include evaluating serum cytokine levels, evaluating tumor biopsies for expression of suppressive factors such as IDO, or evaluating the methylation status of the Foxp3 promoter as a surrogate for Treg activity (Polansky et al., 2008; Wieczorek et al., 2009).

A fairly comprehensive clinical characterization of regulatory T cell activity was reported by François and colleagues, in which melanoma patients were immunized with a MHC class II peptide (Francois et al., 2009). Patient PBMC samples were sorted for CD4+Tetramer+ cells, which were then cloned by limiting dilution and expanded. They found that 5% of these CD4+ T-cell clones also expressed CD25 and Foxp3, and were able to suppress the proliferation of naïve T cells *in vitro* as well as the secretion of IFNγ, IL-2, IL-10, and TNFα by effector T cells. These CD4+CD25+Foxp3+ T-cell clones expressed TGF-β, and had unmethylated Foxp3 promoters, consistent with the patterns observed in Tregs (Polansky et al., 2008). While this report did not observe any correlations between Treg frequency and responses to immunization, it exemplifies the type of functional analysis that can shed light on Treg activity.

As a measure of antigen-specific regulation not requiring *in vitro* amplification, an alternative methodology that can be used is the trans vivo delayed-type hypersensitivity (tvDTH) assay. This assay has been commonly used to evaluate antigen-specific tolerance in transplant recipients, but has rarely been used to evaluate regulation in tumor immunotherapy clinical trials (Carrodeguas et al., 1999; VanBuskirk et al., 2000; Cai et al., 2004; Olson et al., 2012). For this assay, peripheral blood samples from patients are injected into the foodpads of SCID mice along with a recall antigen (such as tetanus toxoid or inactivated Epstein-Barr virus) and an experimental antigen being evaluated for regulatory immune responses. As with other antigen-specific regulatory immune responses, the experimental antigen-specific regulatory cells require their cognate antigen for activation, but once activated can suppress in a non-specific fashion (thus suppressing the tetanus bystander immune response). Twenty-four hours later, footpad swelling can be measured as an indicator of an inflammatory immune response, which can be suppressed by the activation of antigen-specific regulatory responses. Thus, this assay does not rely on measuring any single metric of suppressive activity, but rather measures how all of these mechanisms act in concert to suppress inflammation *in vivo*. However, by including blocking antibodies specific to particular suppressive functions (such as antibodies blocking TGF-β, IL-10, or IL-35, or surface molecules such as CTLA-4 or PD-1), it is possible to identify mechanisms that contribute to suppression. Furthermore, by conducting the assay with particular T-cell subsets, it is possible to identify the cell population that mediates suppression.

The tvDTH assay is also useful in that it can be used to detect effector responses that are suppressed by concurrent regulatory responses. As we have described in a report evaluating a DNA vaccine in patients with prostate cancer, we found that we were not able to detect antigen-specific effector responses in peripheral blood samples from multiple patients when the antigen of interest was injected into the footpads of SCID mice alone. However, when we blocked regulatory responses using antibodies specific to CTLA-4, we were able to uncover antigen-specific effector responses that were otherwise undetectable (Olson et al., 2012). These suppressed effector responses can also be detected using standard *in vitro* techniques using peripheral blood samples that have been depleted of regulatory cells, as has been reported – however, these assays still only measure particular aspects of Treg activity (Gnjatic et al., 2009; Hadaschik et al., 2012). However, the masking of effector responses by concurrent regulatory illustrates one of the most important reasons to include analysis of regulatory populations in clinical trials evaluating immunotherapies; given the interaction between effector and regulatory populations, it is possible that ignoring one population could compromise the analysis of the other.

## Concluding Remarks

As immunotherapies for the treatment of cancer begin to show clinical benefit and become approved for use in the clinic, it is of crucial importance to identify biomarkers of efficacy that can also be incorporated into the clinic, both to identify which patients may optimally respond to therapy, as well as to determine whether individual patients are responding to therapy. This is particularly relevant because most immunotherapy clinical trials have relied on measuring overall survival as a primary clinical endpoint, and while overall survival remains the gold-standard for determining clinical efficacy, it is an impractical endpoint for making clinical treatment decisions. In addition, many treatments rely on repetitive administration (vaccines, for example), and it is conceivable that tolerance/regulation may be elicited that can prevent the generation of productive anti-tumor effector responses. As such, it is critical to monitor for these regulatory responses to determine if/when additional booster immunizations or other immune-modulating agents should be employed.

As our understanding of immune suppression has expanded from the identification of regulatory cell populations to current research aimed at elucidating the plastic nature of immune function and the interplay between effector and regulatory immunity within the tumor microenvironment, it has become evident that regulatory cells play a central role in the development and progression of cancer, and can influence the outcome of tumor immunotherapies. Therefore, it is important to include analysis of these regulatory populations in the immune monitoring of clinical trials, as it can complete the picture of how immune responses are affected by immunotherapeutic intervention. Furthermore, as our understanding of how these regulatory responses are affected by immunization develops, it will be possible to design more optimal combinatorial approaches that seek to activate effector responses as well as inhibit or deplete these suppressive cells.

However, as our understanding of regulatory cells continues to expand, it is important that immune monitoring efforts tracking these cell populations continue to grow as well to address the current challenges associated with the monitoring of regulatory populations. This includes identifying combinations of phenotypic markers that can be used to more reliably track suppressive populations, or alternatively using multiple definitions of regulatory cells to confirm results obtained by examining a single phenotype. It will also be important to incorporate the use of functional analysis of regulatory cell function as is done with effector cells, as this can provide a more complete picture of both the quantity and quality of suppressive responses. Additionally, as our knowledge of the plastic nature of suppressive activity in nominally non-regulatory immune cells expands, it will be important to incorporate this information into immune monitoring, which can help expand this monitoring from defining a single moment in time to generating a more complete understanding of the suppressive potential of the immune response following immunotherapeutic intervention. Equally important, it will be crucial to gain a better understanding for how pre-existing regulatory responses affect the ability to respond to immunotherapy, as this can be used to prospectively identify individuals who are most likely to respond to therapy.

## Conflict of Interest Statement

The authors declare that the research was conducted in the absence of any commercial or financial relationships that could be construed as a potential conflict of interest.

## Abbreviations

CTLA-4, cytotoxic T lymphocyte-associated antigen 4; DC, dendritic cell; ELISA, enzyme-linked immunosorbent assay; ELISPOT, enzyme-linked immunosorbent spot; GM-CSF, granulocyte macrophage colony-stimulating factor; ICCS, intracellular cytokine staining; IDO, indoleamine 2,3-dioxygenase; IL, interleukin; iNOS, inducible nitric oxide synthase; iTreg, induced Treg; MDSC, myeloid-derived suppressor cell; NO, nitric oxide; nTreg, natural Treg; PAP, prostatic acid phosphatase; PBMC, peripheral blood mononuclear cells; ROS, reactive oxygen species; TAM, tumor-associated macrophage; TGF, Transforming growth factor; Treg, Regulatory T cell; tvDTH, trans vivo delayed-type hypersensitivity.

## References

[B1] Addey C. White M. Dou L. Coe D. Dyson J. Chai J. G. (2011). Functional plasticity of antigen-specific regulatory T cells in context of tumor. J. Immunol. 186, 4557–4564 10.4049/jimmunol.1003797 21389255

[B2] Akin Y. Koksoy S. Yucel S. Erdogru T. Baykara M. (2011). Increased peripheral CD4+CD25high Treg in prostate cancer patients is correlated with PSA. Saudi Med. J. 32, 1003–1008 22008917

[B3] Andersen M. H. Sorensen R. B. Brimnes M. K. Svane I. M. Becker J. C. Thor Straten P. (2009). Identification of heme oxygenase-1-specific regulatory CD8+ T cells in cancer patients. J. Clin. Invest. 119, 2245–2256 10.1172/JCI38739 19662679 PMC2719937

[B4] Berntsen A. Brimnes M. K. Thor Straten P. Svane I. M. (2010). Increase of circulating CD4+CD25highFoxp3+ regulatory T cells in patients with metastatic renal cell carcinoma during treatment with dendritic cell vaccination and low-dose interleukin-2. J. Immunother. 33, 425–434 10.1097/CJI.0b013e3181cd870f 20386464

[B5] Betts G. J. Clarke S. L. Richards H. E. Godkin A. J. Gallimore A. M. (2006). Regulating the immune response to tumours. Adv. Drug Deliv. Rev. 58, 948–961 10.1016/j.addr.2006.05.006 17070961

[B6] Beyer M. Kochanek M. Darabi K. Popov A. Jensen M. Endl E. (2005). Reduced frequencies and suppressive function of CD4+CD25hi regulatory T cells in patients with chronic lymphocytic leukemia after therapy with fludarabine. Blood 106, 2018–2025 10.1182/blood-2005-02-0642 15914560

[B7] Biswas S. K. Mantovani A. (2010). Macrophage plasticity and interaction with lymphocyte subsets: cancer as a paradigm. Nat. Immunol. 11, 889–896 10.1038/ni.1937 20856220

[B8] Bjoern J. Brimnes M. K. Andersen M. H. Thor Straten P. Svane I. M. (2011). Changes in peripheral blood level of regulatory T cells in patients with malignant melanoma during treatment with dendritic cell vaccination and low-dose IL-2. Scand. J. Immunol. 73, 222–233 10.1111/j.1365-3083.2010.02494.x 21204893

[B9] Bluestone J. A. Mackay C. R. O’Shea J. J. Stockinger B. (2009). The functional plasticity of T cell subsets. Nat. Rev. Immunol. 9, 811–816 10.1038/nri2654 19809471 PMC3075537

[B10] Bonertz A. Weitz J. Pietsch D. H. Rahbari N. N. Schlude C. Ge Y. (2009). Antigen-specific Tregs control T cell responses against a limited repertoire of tumor antigens in patients with colorectal carcinoma. J. Clin. Invest. 119, 3311–3321 19809157 10.1172/JCI39608 PMC2769188

[B11] Bottazzi B. Polentarutti N. Acero R. Balsari A. Boraschi D. Ghezzi P. (1983). Regulation of the macrophage content of neoplasms by chemoattractants. Science 220, 210–212 10.1126/science.6828888 6828888

[B12] Bronte V. Zanovello P. (2005). Regulation of immune responses by l-arginine metabolism. Nat. Rev. Immunol. 5, 641–654 10.1038/nri1668 16056256

[B13] Cai J. Lee J. Jankowska-Gan E. Derks R. Pool J. Mutis T. (2004). Minor H antigen HA-1-specific regulator and effector CD8+ T cells, and HA-1 microchimerism, in allograft tolerance. J. Exp. Med. 199, 1017–1023 10.1084/jem.20031012 15067036 PMC2211880

[B14] Carrodeguas L. Orosz C. G. Waldman W. J. Sedmak D. D. Adams P. W. Vanbuskirk A. M. (1999). Trans vivo analysis of human delayed-type hypersensitivity reactivity. Hum. Immunol. 60, 640–651 10.1016/S0198-8859(99)00002-6 10439310

[B15] Chakraborty N. G. Chattopadhyay S. Mehrotra S. Chhabra A. Mukherji B. (2004). Regulatory T-cell response and tumor vaccine-induced cytotoxic T lymphocytes in human melanoma. Hum. Immunol. 65, 794–802 10.1016/j.humimm.2004.05.012 15336780

[B16] Chaput N. Louafi S. Bardier A. Charlotte F. Vaillant J. C. Menegaux F. (2009). Identification of CD8+CD25+Foxp3+ suppressive T cells in colorectal cancer tissue. Gut 58, 520–529 10.1136/gut.2008.158824 19022917

[B17] Chen Y. Kuchroo V. K. Inobe J. Hafler D. A. Weiner H. L. (1994). Regulatory T cell clones induced by oral tolerance: suppression of autoimmune encephalomyelitis. Science 265, 1237–1240 10.1126/science.8016656 7520605

[B18] Coffelt S. B. Hughes R. Lewis C. E. (2009). Tumor-associated macrophages: effectors of angiogenesis and tumor progression. Biochim. Biophys. Acta 1796, 11–18 19269310 10.1016/j.bbcan.2009.02.004

[B19] Collison L. W. Chaturvedi V. Henderson A. L. Giacomin P. R. Guy C. Bankoti J. (2010). IL-35-mediated induction of a potent regulatory T cell population. Nat. Immunol. 11, 1093–1101 10.1038/ni.1952 20953201 PMC3008395

[B20] Collison L. W. Workman C. J. Kuo T. T. Boyd K. Wang Y. Vignali K. M. (2007). The inhibitory cytokine IL-35 contributes to regulatory T-cell function. Nature 450, 566–569 10.1038/nature06306 18033300

[B21] Correale P. Rotundo M. S. Del Vecchio M. T. Remondo C. Migali C. Ginanneschi C. (2010). Regulatory (FoxP3+) T-cell tumor infiltration is a favorable prognostic factor in advanced colon cancer patients undergoing chemo or chemoimmunotherapy. J. Immunother. 33, 435–441 10.1097/CJI.0b013e3181d32f01 20386463 PMC7322625

[B22] Cortesini R. Lemaoult J. Ciubotariu R. Cortesini N. S. (2001). CD8+CD28− T suppressor cells and the induction of antigen-specific, antigen-presenting cell-mediated suppression of Th reactivity. Immunol. Rev. 182, 201–206 10.1034/j.1600-065X.2001.1820116.x 11722635

[B23] Curiel T. J. Coukos G. Zou L. Alvarez X. Cheng P. Mottram P. (2004). Specific recruitment of regulatory T cells in ovarian carcinoma fosters immune privilege and predicts reduced survival. Nat. Med. 10, 942–949 10.1038/nm1093 15322536

[B24] Di Nicola M. Zappasodi R. Carlo-Stella C. Mortarini R. Pupa S. M. Magni M. (2009). Vaccination with autologous tumor-loaded dendritic cells induces clinical and immunologic responses in indolent B-cell lymphoma patients with relapsed and measurable disease: a pilot study. Blood 113, 18–27 10.1182/blood-2008-06-165654 18809757

[B25] Finkelstein S. E. Iclozan C. Bui M. M. Cotter M. J. Ramakrishnan R. Ahmed J. (2012). Combination of external beam radiotherapy (EBRT) with intratumoral injection of dendritic cells as neo-adjuvant treatment of high-risk soft tissue sarcoma patients. Int. J. Radiat. Oncol. Biol. Phys. 82, 924–932 10.1016/j.ijrobp.2010.12.068 21398051 PMC4241354

[B26] Fong B. Jin R. Wang X. Safaee M. Lisiero D. N. Yang I. (2012). Monitoring of regulatory T cell frequencies and expression of CTLA-4 on T cells, before and after DC vaccination, can predict survival in GBM patients. PLoS ONE 7:e32614 10.1371/journal.pone.0032614 22485134 PMC3317661

[B27] Francois V. Ottaviani S. Renkvist N. Stockis J. Schuler G. Thielemans K. (2009). The CD4(+) T-cell response of melanoma patients to a MAGE-A3 peptide vaccine involves potential regulatory T cells. Cancer Res. 69, 4335–4345 10.1158/0008-5472.CAN-08-3726 19435913

[B28] Gabrilovich D. I. Nagaraj S. (2009). Myeloid-derived suppressor cells as regulators of the immune system. Nat. Rev. Immunol. 9, 162–174 10.1038/nri2506 19197294 PMC2828349

[B29] Gates J. D. Clifton G. T. Benavides L. C. Sears A. K. Carmichael M. G. Hueman M. T. (2010). Circulating regulatory T cells (CD4+CD25+FOXP3+) decrease in breast cancer patients after vaccination with a modified MHC class II HER2/neu (AE37) peptide. Vaccine 28, 7476–7482 10.1016/j.vaccine.2010.09.029 20858449

[B30] Gershon R. K. Kondo K. (1970). Cell interactions in the induction of tolerance: the role of thymic lymphocytes. Immunology 18, 723–737 4911896 PMC1455602

[B31] Gershon R. K. Kondo K. (1971). Infectious immunological tolerance. Immunology 21, 903–914 4943147 PMC1408252

[B32] Gnjatic S. Altorki N. K. Tang D. N. Tu S. M. Kundra V. Ritter G. (2009). NY-ESO-1 DNA vaccine induces T-cell responses that are suppressed by regulatory T cells. Clin. Cancer Res. 15, 2130–2139 10.1158/1078-0432.CCR-08-2632 19276258 PMC5806520

[B33] Groux H. O’Garra A. Bigler M. Rouleau M. Antonenko S. De Vries J. E. (1997). A CD4+ T-cell subset inhibits antigen-specific T-cell responses and prevents colitis. Nature 389, 737–742 10.1038/39614 9338786

[B34] Gulley J. L. Arlen P. M. Madan R. A. Tsang K. Y. Pazdur M. P. Skarupa L. (2010). Immunologic and prognostic factors associated with overall survival employing a poxviral-based PSA vaccine in metastatic castrate-resistant prostate cancer. Cancer Immunol. Immunother. 59, 663–674 10.1007/s00262-009-0782-8 19890632 PMC2832083

[B35] Hadaschik B. Su Y. Huter E. Ge Y. Hohenfellner M. Beckhove P. (2012). Antigen specific T-Cell responses against tumor antigens are controlled by regulatory T cells in patients with prostate cancer. J. Urol. 187, 1458–1465 10.1016/j.juro.2011.11.083 22341271

[B36] Hamid O. Schmidt H. Nissan A. Ridolfi L. Aamdal S. Hansson J. (2011). A prospective phase II trial exploring the association between tumor microenvironment biomarkers and clinical activity of ipilimumab in advanced melanoma. J. Transl. Med. 9, 204 10.1186/1479-5876-9-204 22123319 PMC3239318

[B37] Heckel M. C. Wolfson A. Slachta C. A. Schwarting R. Salgame P. Katsetos C. D. (2011). Human breast tumor cells express IL-10 and IL-12p40 transcripts and proteins, but do not produce IL-12p70. Cell. Immunol. 266, 143–153 10.1016/j.cellimm.2010.09.010 21055733

[B38] Heusinkveld M. van der Burg S. H. (2011). Identification and manipulation of tumor associated macrophages in human cancers. J. Transl. Med. 9, 216 10.1186/1479-5876-9-216 22176642 PMC3286485

[B39] Hiraoka N. Onozato K. Kosuge T. Hirohashi S. (2006). Prevalence of FOXP3+ regulatory T cells increases during the progression of pancreatic ductal adenocarcinoma and its premalignant lesions. Clin. Cancer Res. 12, 5423–5434 10.1158/1078-0432.CCR-06-0369 17000676

[B40] Hodi F. S. O’Day S. J. Mcdermott D. F. Weber R. W. Sosman J. A. Haanen J. B. (2010). Improved survival with ipilimumab in patients with metastatic melanoma. N. Engl. J. Med. 363, 711–723 10.1056/NEJMoa1003466 20525992 PMC3549297

[B41] Huang B. Pan P. Y. Li Q. Sato A. I. Levy D. E. Bromberg J. (2006). Gr-1+CD115+ immature myeloid suppressor cells mediate the development of tumor-induced T regulatory cells and T-cell anergy in tumor-bearing host. Cancer Res. 66, 1123–1131 10.1158/0008-5472.CAN-06-0077 16424049

[B42] Hus I. Schmitt M. Tabarkiewicz J. Radej S. Wojas K. Bojarska-Junak A. (2008). Vaccination of B-CLL patients with autologous dendritic cells can change the frequency of leukemia antigen-specific CD8+ T cells as well as CD4+CD25+FoxP3+ regulatory T cells toward an antileukemia response. Leukemia 22, 1007–1017 10.1038/leu.2008.29 18323802

[B43] Jordan M. S. Boesteanu A. Reed A. J. Petrone A. L. Holenbeck A. E. Lerman M. A. (2001). Thymic selection of CD4+CD25+ regulatory T cells induced by an agonist self-peptide. Nat. Immunol. 2, 301–306 10.1038/86302 11276200

[B44] Kantoff P. W. Higano C. S. Shore N. D. Berger E. R. Small E. J. Penson D. F. (2010). Sipuleucel-T immunotherapy for castration-resistant prostate cancer. N. Engl. J. Med. 363, 411–422 10.1056/NEJMoa1001294 20818862

[B45] Katz S. C. Bamboat Z. M. Maker A. V. Shia J. Pillarisetty V. G. Yopp A. C. (2013). Regulatory T cell infiltration predicts outcome following resection of colorectal cancer liver metastases. Ann. Surg. Oncol. 20, 946–955 10.1245/s10434-012-2668-9 23010736 PMC3740360

[B46] Kavanagh B. O’Brien S. Lee D. Hou Y. Weinberg V. Rini B. (2008). CTLA4 blockade expands FoxP3+ regulatory and activated effector CD4+ T cells in a dose-dependent fashion. Blood 112, 1175–1183 10.1182/blood-2007-11-125435 18523152 PMC2515138

[B47] Kiniwa Y. Miyahara Y. Wang H. Y. Peng W. Peng G. Wheeler T. M. (2007). CD8+ Foxp3+ regulatory T cells mediate immunosuppression in prostate cancer. Clin. Cancer Res. 13, 6947–6958 10.1158/1078-0432.CCR-07-0842 18056169

[B48] Kobayashi N. Hiraoka N. Yamagami W. Ojima H. Kanai Y. Kosuge T. (2007). FOXP3+ regulatory T cells affect the development and progression of hepatocarcinogenesis. Clin. Cancer Res. 13, 902–911 10.1158/1078-0432.CCR-06-2730 17289884

[B49] Kono K. Kawaida H. Takahashi A. Sugai H. Mimura K. Miyagawa N. (2006). CD4(+)CD25high regulatory T cells increase with tumor stage in patients with gastric and esophageal cancers. Cancer Immunol. Immunother. 55, 1064–1071 10.1007/s00262-005-0092-8 16328385 PMC11030626

[B50] Kusmartsev S. Nefedova Y. Yoder D. Gabrilovich D. I. (2004). Antigen-specific inhibition of CD8+ T cell response by immature myeloid cells in cancer is mediated by reactive oxygen species. J. Immunol. 172, 989–999 14707072 10.4049/jimmunol.172.2.989

[B51] Lehe C. Ghebeh H. Al-Sulaiman A. Al Qudaihi G. Al-Hussein K. Almohareb F. (2008). The Wilms’ tumor antigen is a novel target for human CD4+ regulatory T cells: implications for immunotherapy. Cancer Res. 68, 6350–6359 10.1158/0008-5472.CAN-08-0050 18676860

[B52] Levings M. K. Gregori S. Tresoldi E. Cazzaniga S. Bonini C. Roncarolo M. G. (2005). Differentiation of Tr1 cells by immature dendritic cells requires IL-10 but not CD25+CD4+ Tr cells. Blood 105, 1162–1169 10.1182/blood-2004-03-1211 15479730

[B53] Liakou C. I. Kamat A. Tang D. N. Chen H. Sun J. Troncoso P. (2008). CTLA-4 blockade increases IFNgamma-producing CD4+ICOShi cells to shift the ratio of effector to regulatory T cells in cancer patients. Proc. Natl. Acad. Sci. U.S.A. 105, 14987–14992 10.1073/pnas.0806075105 18818309 PMC2567480

[B54] Lissoni P. Brivio F. Fumagalli L. Messina G. Meregalli S. Porro G. (2009). Effects of the conventional antitumor therapies surgery, chemotherapy, radiotherapy and immunotherapy on regulatory T lymphocytes in cancer patients. Anticancer Res. 29, 1847–1852 19443415

[B55] Liu V. C. Wong L. Y. Jang T. Shah A. H. Park I. Yang X. (2007). Tumor evasion of the immune system by converting CD4+CD25− T cells into CD4+CD25+ T regulatory cells: role of tumor-derived TGF-beta. J. Immunol. 178, 2883–2892 17312132 10.4049/jimmunol.178.5.2883

[B56] Liyanage U. K. Moore T. T. Joo H. G. Tanaka Y. Herrmann V. Doherty G. (2002). Prevalence of regulatory T cells is increased in peripheral blood and tumor microenvironment of patients with pancreas or breast adenocarcinoma. J. Immunol. 169, 2756–2761 12193750 10.4049/jimmunol.169.5.2756

[B57] Lohr J. Knoechel B. Abbas A. K. (2006). Regulatory T cells in the periphery. Immunol. Rev. 212, 149–162 10.1111/j.0105-2896.2006.00414.x 16903912

[B58] Mantovani A. Sozzani S. Locati M. Allavena P. Sica A. (2002). Macrophage polarization: tumor-associated macrophages as a paradigm for polarized M2 mononuclear phagocytes. Trends Immunol. 23, 549–555 10.1016/S1471-4906(02)02302-5 12401408

[B59] Mazzoni A. Bronte V. Visintin A. Spitzer J. H. Apolloni E. Serafini P. (2002). Myeloid suppressor lines inhibit T cell responses by an NO-dependent mechanism. J. Immunol. 168, 689–695 11777962 10.4049/jimmunol.168.2.689

[B60] Mellor A. L. Munn D. H. (2004). IDO expression by dendritic cells: tolerance and tryptophan catabolism. Nat. Rev. Immunol. 4, 762–774 10.1038/nri1457 15459668

[B61] Miller A. M. Lundberg K. Ozenci V. Banham A. H. Hellstrom M. Egevad L. (2006). CD4+CD25high T cells are enriched in the tumor and peripheral blood of prostate cancer patients. J. Immunol. 177, 7398–7405 17082659 10.4049/jimmunol.177.10.7398

[B62] Montero A. J. Diaz-Montero C. M. Kyriakopoulos C. E. Bronte V. Mandruzzato S. (2012). Myeloid-derived suppressor cells in cancer patients: a clinical perspective. J. Immunother. 35, 107–115 10.1097/CJI.0b013e318242169f 22306898

[B63] Mougiakakos D. Choudhury A. Lladser A. Kiessling R. Johansson C. C. (2010). Regulatory T cells in cancer. Adv. Cancer Res. 107, 57–117 10.1016/S0065-230X(10)07003-X 20399961

[B64] Movahedi K. Guilliams M. Van Den Bossche J. Van Den Bergh R. Gysemans C. Beschin A. (2008). Identification of discrete tumor-induced myeloid-derived suppressor cell subpopulations with distinct T cell-suppressive activity. Blood 111, 4233–4244 10.1182/blood-2007-07-099226 18272812

[B65] Nagaraj S. Gupta K. Pisarev V. Kinarsky L. Sherman S. Kang L. (2007). Altered recognition of antigen is a mechanism of CD8+ T cell tolerance in cancer. Nat. Med. 13, 828–835 10.1038/nm1609 17603493 PMC2135607

[B66] Olson B. M. Jankowska-Gan E. Becker J. T. Vignali D. A. Burlingham W. J. Mcneel D. G. (2012). Human prostate tumor antigen-specific CD8+ regulatory T cells are inhibited by CTLA-4 or IL-35 blockade. J. Immunol. 189, 5590–5601 10.4049/jimmunol.1201744 23152566 PMC3735346

[B67] O’Mahony D. Morris J. C. Quinn C. Gao W. Wilson W. H. Gause B. (2007). A pilot study of CTLA-4 blockade after cancer vaccine failure in patients with advanced malignancy. Clin. Cancer Res. 13, 958–964 10.1158/1078-0432.CCR-06-1974 17289891

[B68] Pages F. Galon J. Dieu-Nosjean M. C. Tartour E. Sautes-Fridman C. Fridman W. H. (2010). Immune infiltration in human tumors: a prognostic factor that should not be ignored. Oncogene 29, 1093–1102 10.1038/onc.2009.416 19946335

[B69] Peranzoni E. Zilio S. Marigo I. Dolcetti L. Zanovello P. Mandruzzato S. (2010). Myeloid-derived suppressor cell heterogeneity and subset definition. Curr. Opin. Immunol. 22, 238–244 10.1016/j.coi.2010.01.021 20171075

[B70] Pohla H. Buchner A. Stadlbauer B. Frankenberger B. Stevanovic S. Walter S. (2013). High immune response rates and decreased frequencies of regulatory T cells in metastatic renal cell carcinoma patients after tumor cell vaccination. Mol. Med. 18, 1499–1508 23269976 10.2119/molmed.2012.00221 PMC3576476

[B71] Polansky J. K. Kretschmer K. Freyer J. Floess S. Garbe A. Baron U. (2008). DNA methylation controls Foxp3 gene expression. Eur. J. Immunol. 38, 1654–1663 10.1002/eji.200838105 18493985

[B72] Prins R. M. Wang X. Soto H. Young E. Lisiero D. N. Fong B. (2013). Comparison of glioma-associated antigen peptide-loaded versus autologous tumor lysate-loaded dendritic cell vaccination in malignant glioma patients. J. Immunother. 36, 152–157 10.1097/CJI.0b013e3182811ae4 23377664 PMC3568250

[B73] Qin S. Cobbold S. P. Pope H. Elliott J. Kioussis D. Davies J. (1993). “Infectious” transplantation tolerance. Science 259, 974–977 10.1126/science.8094901 8094901

[B74] Read S. Malmstrom V. Powrie F. (2000). Cytotoxic T lymphocyte-associated antigen 4 plays an essential role in the function of CD25(+)CD4(+) regulatory cells that control intestinal inflammation. J. Exp. Med. 192, 295–302 10.1084/jem.192.2.295 10899916 PMC2193261

[B75] Roncarolo M. G. Bacchetta R. Bordignon C. Narula S. Levings M. K. (2001). Type 1 T regulatory cells. Immunol. Rev. 182, 68–79 10.1034/j.1600-065X.2001.1820105.x 11722624

[B76] Santegoets S. J. Stam A. G. Lougheed S. M. Gall H. Scholten P. E. Reijm M. (2013). T cell profiling reveals high CD4(+)CTLA-4 (+) T cell frequency as dominant predictor for survival after Prostate GVAX/ipilimumab treatment. Cancer Immunol. Immunother. 62, 245–256 10.1007/s00262-012-1330-5 22878899 PMC11029684

[B77] Sarantopoulos S. Lu L. Cantor H. (2004). Qa-1 restriction of CD8+ suppressor T cells. J. Clin. Invest. 114, 1218–1221 10.1172/JCI200423152 15520850 PMC524234

[B78] Schwarzer A. Wolf B. Fisher J. L. Schwaab T. Olek S. Baron U. (2012). Regulatory T-cells and associated pathways in metastatic renal cell carcinoma (mRCC) patients undergoing DC-vaccination and cytokine-therapy. PLoS ONE 7:e46600 10.1371/journal.pone.0046600 23118856 PMC3485261

[B79] Serafini P. Mgebroff S. Noonan K. Borrello I. (2008). Myeloid-derived suppressor cells promote cross-tolerance in B-cell lymphoma by expanding regulatory T cells. Cancer Res. 68, 5439–5449 10.1158/0008-5472.CAN-07-6621 18593947 PMC2887390

[B80] Shafer-Weaver K. A. Anderson M. J. Stagliano K. Malyguine A. Greenberg N. M. Hurwitz A. A. (2009). Cutting edge: tumor-specific CD8+ T cells infiltrating prostatic tumors are induced to become suppressor cells. J. Immunol. 183, 4848–4852 10.4049/jimmunol.0900848 19801511 PMC6948842

[B81] Takahashi T. Tagami T. Yamazaki S. Uede T. Shimizu J. Sakaguchi N. (2000). Immunologic self-tolerance maintained by CD25(+)CD4(+) regulatory T cells constitutively expressing cytotoxic T lymphocyte-associated antigen 4. J. Exp. Med. 192, 303–310 10.1084/jem.192.2.303 10899917 PMC2193248

[B82] Tarhini A. Edington H. Butterfield L. Tawbi H. Moschos S. Shuai Y. (2012). “Neoadjuvant ipilimumab in locally/regionally advanced melanoma: clinical outcome and immune monitoring,” in 2012 ASCO Annual Meeting, Chicago, Abstract 8533.

[B83] Valzasina B. Piconese S. Guiducci C. Colombo M. P. (2006). Tumor-induced expansion of regulatory T cells by conversion of CD4+CD25− lymphocytes is thymus and proliferation independent. Cancer Res. 66, 4488–4495 10.1158/0008-5472.CAN-05-4217 16618776

[B84] van der Burg S. H. Piersma S. J. De Jong A. Van Der Hulst J. M. Kwappenberg K. M. Van Den Hende M. (2007). Association of cervical cancer with the presence of CD4+ regulatory T cells specific for human papillomavirus antigens. Proc. Natl. Acad. Sci. U.S.A. 104, 12087–12092 10.1073/pnas.0704672104 17615234 PMC1924590

[B85] VanBuskirk A. M. Burlingham W. J. Jankowska-Gan E. Chin T. Kusaka S. Geissler F. (2000). Human allograft acceptance is associated with immune regulation. J. Clin. Invest. 106, 145–155 10.1172/JCI9171 10880058 PMC314359

[B86] Vasiljeva O. Papazoglou A. Kruger A. Brodoefel H. Korovin M. Deussing J. (2006). Tumor cell-derived and macrophage-derived cathepsin B promotes progression and lung metastasis of mammary cancer. Cancer Res. 66, 5242–5250 10.1158/0008-5472.CAN-05-4463 16707449

[B87] Vence L. Palucka A. K. Fay J. W. Ito T. Liu Y. J. Banchereau J. (2007). Circulating tumor antigen-specific regulatory T cells in patients with metastatic melanoma. Proc. Natl. Acad. Sci. U.S.A. 104, 20884–20889 10.1073/pnas.0710557105 18093940 PMC2409236

[B88] Vergati M. Cereda V. Madan R. A. Gulley J. L. Huen N. Y. Rogers C. J. (2011). Analysis of circulating regulatory T cells in patients with metastatic prostate cancer pre- versus post-vaccination. Cancer Immunol. Immunother. 60, 197–206 10.1007/s00262-010-0927-9 20976449 PMC3202216

[B89] Vignali D. A. Collison L. W. Workman C. J. (2008). How regulatory T cells work. Nat. Rev. Immunol. 8, 523–532 10.1038/nri2343 18566595 PMC2665249

[B90] von Herrath M. G. Harrison L. C. (2003). Antigen-induced regulatory T cells in autoimmunity. Nat. Rev. Immunol. 3, 223–232 10.1038/nri1029 12658270

[B91] Wang H. Y. Lee D. A. Peng G. Guo Z. Li Y. Kiniwa Y. (2004). Tumor-specific human CD4+ regulatory T cells and their ligands: implications for immunotherapy. Immunity 20, 107–118 10.1016/S1074-7613(04)00030-5 14738769

[B92] Wang H. Y. Peng G. Guo Z. Shevach E. M. Wang R. F. (2005). Recognition of a new ARTC1 peptide ligand uniquely expressed in tumor cells by antigen-specific CD4+ regulatory T cells. J. Immunol. 174, 2661–2670 15728473 10.4049/jimmunol.174.5.2661

[B93] Wang Z. Liu J. Q. Liu Z. Shen R. Zhang G. Xu J. (2013). Tumor-derived IL-35 promotes tumor growth by enhancing myeloid cell accumulation and angiogenesis. J. Immunol. 190, 2415–2423 10.4049/jimmunol.1202535 23345334 PMC3578001

[B94] Wei S. Kryczek I. Zou L. Daniel B. Cheng P. Mottram P. (2005). Plasmacytoid dendritic cells induce CD8+ regulatory T cells in human ovarian carcinoma. Cancer Res. 65, 5020–5026 10.1158/0008-5472.CAN-05-0716 15958543

[B95] Weiner H. L. (2001). Induction and mechanism of action of transforming growth factor-beta-secreting Th3 regulatory cells. Immunol. Rev. 182, 207–214 10.1034/j.1600-065X.2001.1820117.x 11722636

[B96] Welters M. J. Kenter G. G. Piersma S. J. Vloon A. P. Lowik M. J. Berends-Van Der Meer D. M. (2008). Induction of tumor-specific CD4+ and CD8+ T-cell immunity in cervical cancer patients by a human papillomavirus type 16 E6 and E7 long peptides vaccine. Clin. Cancer Res. 14, 178–187 10.1158/1078-0432.CCR-07-1880 18172269

[B97] Wieczorek G. Asemissen A. Model F. Turbachova I. Floess S. Liebenberg V. (2009). Quantitative DNA methylation analysis of FOXP3 as a new method for counting regulatory T cells in peripheral blood and solid tissue. Cancer Res. 69, 599–608 10.1158/0008-5472.CAN-08-2361 19147574

[B98] Wolf D. Wolf A. M. Rumpold H. Fiegl H. Zeimet A. G. Muller-Holzner E. (2005). The expression of the regulatory T cell-specific forkhead box transcription factor FoxP3 is associated with poor prognosis in ovarian cancer. Clin. Cancer Res. 11, 8326–8331 10.1158/1078-0432.CCR-05-1244 16322292

[B99] Woo E. Y. Chu C. S. Goletz T. J. Schlienger K. Yeh H. Coukos G. (2001). Regulatory CD4(+)CD25(+) T cells in tumors from patients with early-stage non-small cell lung cancer and late-stage ovarian cancer. Cancer Res. 61, 4766–4772 11406550

[B100] Woo E. Y. Yeh H. Chu C. S. Schlienger K. Carroll R. G. Riley J. L. (2002). Cutting edge: regulatory T cells from lung cancer patients directly inhibit autologous T cell proliferation. J. Immunol. 168, 4272–4276 11970966 10.4049/jimmunol.168.9.4272

[B101] Yang R. Cai Z. Zhang Y. Yutzy W. H. T. Roby K. F. Roden R. B. (2006). CD80 in immune suppression by mouse ovarian carcinoma-associated Gr-1+CD11b+ myeloid cells. Cancer Res. 66, 6807–6815 10.1158/0008-5472.CAN-05-0425 16818658

